# Sharper vision, steady hands: can robots improve subretinal drug delivery? Systematic review

**DOI:** 10.1007/s11701-024-01991-x

**Published:** 2024-05-31

**Authors:** Paweł Marek Łajczak, Zbigniew Nawrat

**Affiliations:** 1 Zbigniew Religa Student Scientific Club at Department of Biophysics, Faculty of Medical Sciences in Zabrze, Medical University of Silesia in Katowice, Jordana 18, 40-043 Zabrze, Poland; 2 Department of Biophysics, Faculty of Medical Sciences in Zabrze, Medical University of Silesia in Katowice, Jordana 18, 40-043 Zabrze, Poland; 3 Foundation of Cardiac Surgery Development, 41-808 Zabrze, Poland

**Keywords:** Robot assisted, Subretinal injection, Drug injection, Retina

## Abstract

Subretinal injection (SI) is a novel drug delivery method, directly to retina for treatment of various eye disease. However, manual injection requires surgical experience and precision due to physiological factors. Robots offer solution to this issue, by reducing hand tremor and increased accuracy. This systematic review analyzes current status on robot-assisted SI compared to conventional techniques. Systematic search across 5 databases was conducted to identify studies comparing manual and robot-assisted SI procedures. Extracted data included robotic systems, complications, and success rates. Four studies, including one human trial, two ex vivo porcine eye studies, and one artificial eye model study were included in the synthesis. The findings show early advantages of robot-assisted SI. Compared to traditional interventions, robot procedures result in reduced tremor, what potentially lowers the risk of complications, including retinal tears and reflux. The first in-human randomized trial showed encouraging results, with no significant differences in surgical times or complications between robot-assisted and manual SI. However, major limitation of robot-assisted procedures is longer procedure time. Robot-assisted SI holds promise by offering increased precision and stability, reducing human error and potentially improving clinical outcomes. Challenges include cost, availability, and learning curve. Overall, early stage of robot-assisted SI suggests advantages in precision, complication reduction, and potentially improved drug delivery. Further research in human randomized trials is needed to fully assess its full-scale clinical application.

## Introduction

Robotics is finding increasing applications in ophthalmology, contributing to the precision, safety, and efficiency of medical procedures. The first group of robots is related to diagnostics. Advanced robotics imaging systems, such as optical coherence tomography (OCT), are used to obtain high-resolution images of the eye’s structures. Robots can automatically adjust the positioning of the device and the scanning, which increases the accuracy of the diagnosis. Robots are used for precise shaping of the cornea in procedures aimed at correcting vision defects such as myopia, hyperopia, and astigmatism. Robots can be used to precisely remove the clouded lens of the eye and replace it with an artificial implant. Procedures on the retina, such as the removal of epiretinal membranes or the repair of retinal detachment, require exceptional precision due to the delicacy of the eye’s structures. Surgical robots could perform these operations with greater accuracy than the human hand, which is crucial for optimal results.

Robots can be used for precise administration of intraocular drugs, often used in the treatment of diseases such as age-related macular degeneration or diabetic retinopathy. Robotics ensures accurate dosing and minimizes the risk of infection.

Subretinal injection (SI) is a novel method used to treat various retinal diseases, including genetic defects [1]. SI involves placing a drug solution into the subretinal space, near the photoreceptor and retinal pigment epithelium (RPE) cells. These cells are responsible for nutrition of cones and rods, phagocytosis, regulation of retinal fluids, and build up immunological barrier [2–4]. SI procedure requires a high level of surgical skill and precision, as the surgeon must keep the needle steady for several minutes until the entire volume of the drug is delivered.

The precision required for SI means there are some limitations. Factors like the natural tremor in a human hand and the limited depth resolution when looking through a microscope can affect the accuracy of the SI [5]. If the substance being delivered leaks into the vitreous cavity, it can trigger an immune response [6]. The experience of the surgeon and the operating team greatly influences the success of the surgery. Inaccurate injections can lead to complications such as stretching of the retina, penetration of the choroid, macular holes, retinal detachment, or vitreous hemorrhage [7].

**Fig. 1:**
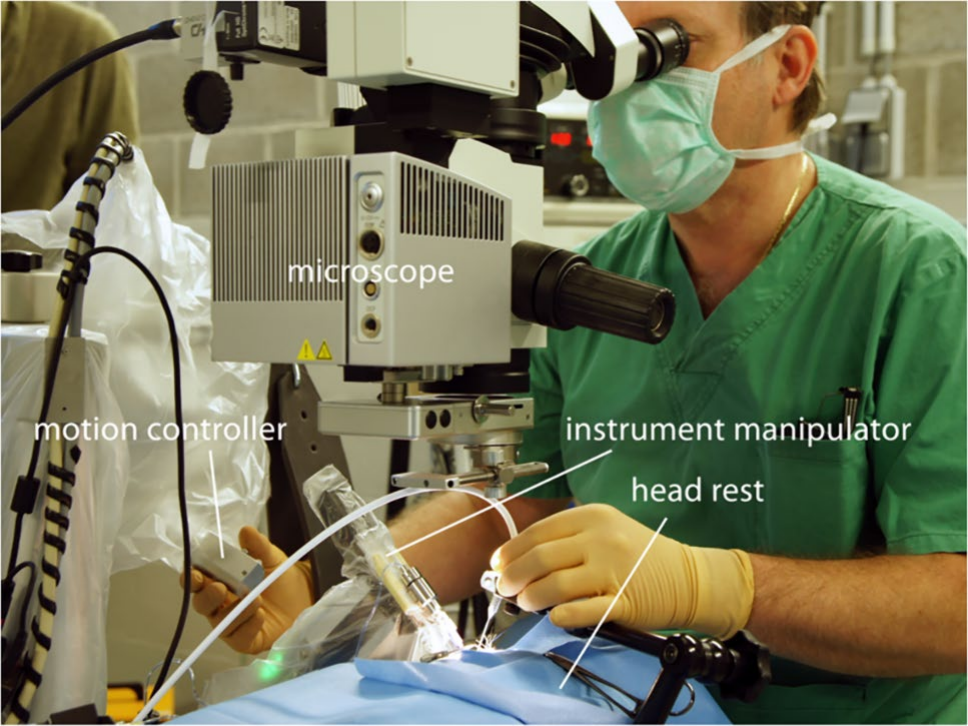
Preceyes robotic system. De Smet et al., published with Creative Commons Attribution License [9]

Technological advancements, such as surgical robots, offer solutions to these challenges. Surgical robots have been used in medicine since the 1980s and allow for precise movements, reduction of tremor, and fatigue resistance. They also provide unique movement types that can enhance surgery. An example in ophthalmology is the Preceyes robotic system [8]. This system includes a motion controller, an instrument manipulator, a computer interface, and a headrest. The control unit allows movement in 4 axes for precise needle movements, with speed controlled by a foot pedal. The system provides real-time 3D monitoring, allowing for full control during surgery. In case of an emergency, instruments can be quickly retracted. The Preceyes robot also features a clutch mechanism, dynamic motion scaling, and tremor filtering – Fig. 1 [9].

According to the manufacturer, system provides surgeons with precision better than 20 µm to position and hold instruments steady for an extended period of time [10]. The robot offers instrument positioning with 4 degrees of freedom. It supports movements up to 80° × 80° × 40 mm and provides 720° of instrument rotation.

Robots will only become widespread in those fields where they prove to be essential due to greater precision, shorter procedure times, and fewer failures and medical complications. Therefore, comparing their use to surgeries conducted with traditional methods constitutes the first criterion to overcome to gain proper interest among doctors, their patients, and the payer of services.

With growing interest in robot-assisted surgical procedures, this systematic review aims to synthesize current knowledge of robot-assisted SI drug delivery procedures, compared to manual techniques. To the best of our knowledge, this is the first systematic synthesis on this topic. We aim not only to analyze the performance of robot-assisted procedures compared to conventional manual ones but also to point out current limitations and future perspectives for this technology, which will certainly revolutionize intraocular surgeries.

## Methodology

This systematic review adhered to the preferred reporting items for systematic reviews and meta-analyses (PRISMA) guidelines for transparent reporting [11]. We searched 5 medical databases: PubMed, Embase, Scopus, Web of Science, and Cochrane Reviews, using MeSH terms like ‘robot’, ‘robot-assisted’, ‘subretinal drug delivery’, and ‘subretinal injection’. No filters for language or publication date were applied. The full search strategy is detailed in the Appendix. Zotero bibliography manager software was used to remove duplicate publications. Search process was visualized with PRISMA flow diagram.

We included original articles from peer-reviewed journals that compared manual and robot-assisted subretinal injections. Results with retinal vein cannulation, where subretinal space was not specifically involved, were also excluded. Given the novelty of the subject, we considered studies on in-human procedures, ex vivo models (e.g., porcine eyes), and other eye-simulating models (e.g., gelatin eye model). We excluded abstract-only works, reviews, editorials, and non-original research. Duplicate results were also removed.

For each study, we extracted data on the country of origin, the ophthalmological robot used, the substance injected, the number of cases in both RA and manual groups, the type of needle for SI, complications, and procedure time. Where available, we also included tremor and drift metrics.

## Results

**Fig. 2:**
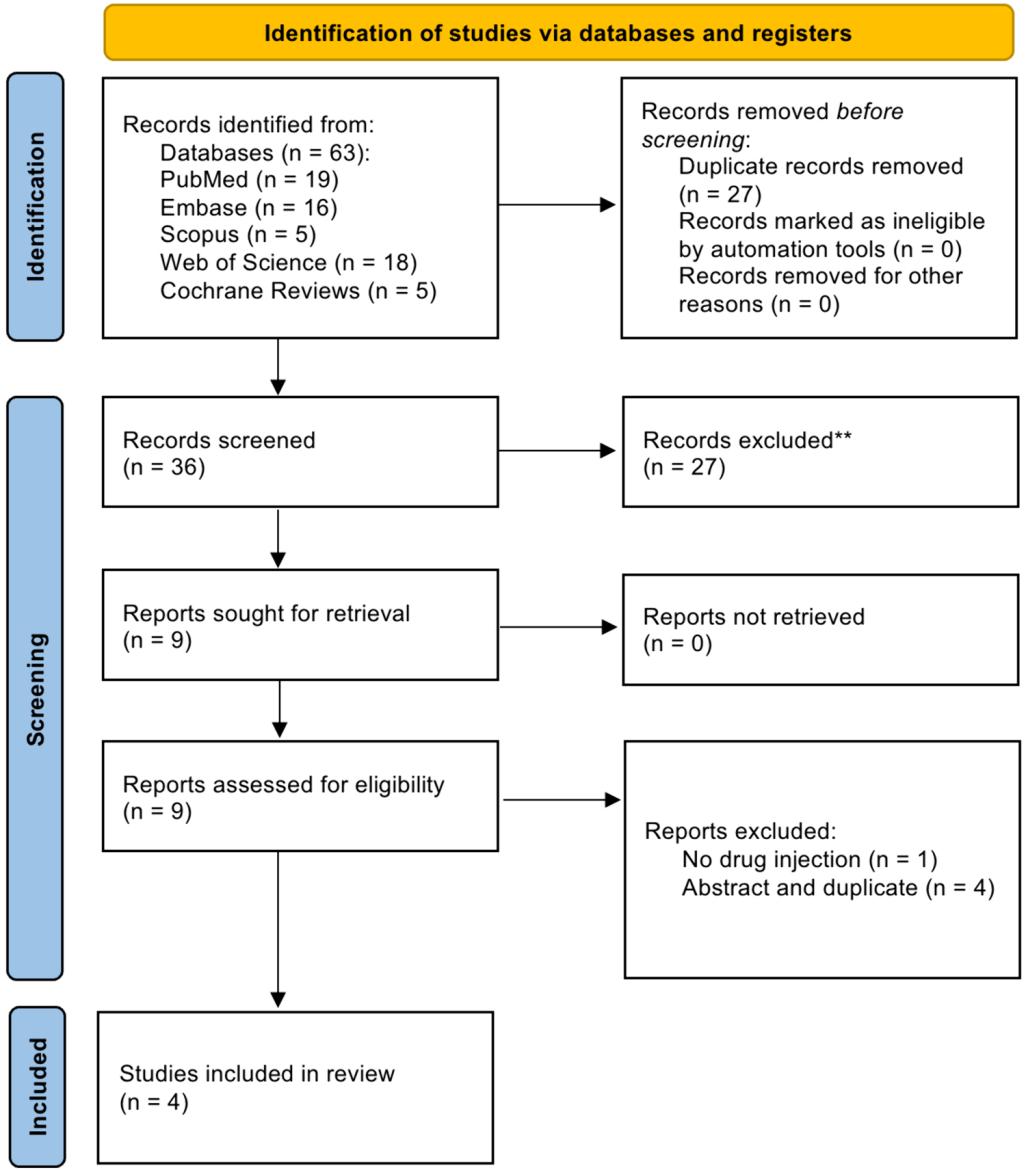
PRISMA flow diagram

The article search was conducted on March 1, 2024, yielding 63 articles: 19 from PubMed, 16 from Embase, 5 from Scopus, 18 from Web of Science, and 5 from Cochrane Reviews. Zotero software identified 27 duplicates for removal. After screening 36 articles, 27 were excluded for irrelevance. Nine underwent eligibility assessment, and 4 were included in this review. Three abstract-only works, one original work (all reporting duplicate results), and one study without drug injection were excluded. The search process is depicted in the PRISMA flow diagram (Fig. 2).

The review included four trials from Belgium, Germany, the UK, and China [5, 12–14]. One was a randomized controlled trial evaluating RA SI in humans. Two assessed efficacies on ex vivo porcine eyes, and one used a gelatin artificial eye model. The studies varied in size, imaging and microscope, needle type, and robotic devices used. The Preceyes surgical system featured in two studies, while the other two utilized custom-built devices. The base characteristics are summarized in Tables 1 and 2.

**Table 1:** Characteristics of included studies

Study	Subject	Needle	Robot	RA: manual cases	Complications in RA group	Complications in manual group
Ladha et al. [5], Belgium 2021	Artificial gelatin eye	38G polyamide	Preceyes Surgical System	9:9	8 bleb formations, 7 refluxes	4 bleb formations, 8 refluxes, 7 puncture hole enlargements
Kapetanovic et al. [12], UK 2021, RCT NCT03052881	Human	41G teflon	Preceyes Surgical System	6:6	1 conversion to manual	None
Maierhofer et al. [13], Germany 2023	Porcine eyes	23G	Custom Robot	38:34	5 refluxes, 4 RPE damage, 1 bleb rupture	3 refluxes, 9 RPE damage, 1 bleb rupture
Yang et al. [14], China 2022	Porcine eyes	41G	Beihang University Custom Robot	5:5	None	None

**Table 2:** Parameters reported from studies

Study	Median injection time	Median tremor	Median drift	Imaging
Ladha et al	Manual: 29 s (range 13–108 s) RA: 52 s (range 18–85 s)	Manual: 18 μm (range 4–266 μm) RA: 1 µm (range 1–11 µm)	Manual: 212 µm (range 115–355 µm) RA: 16 μm (range 4–58 µm)	Operating microscope (OPMI LUMERA 700) with a Resight iOCT (Carl Zeiss Meditec AG, Jena, Germany)
Kapetanovic et al	Manual: 4.3 min (3.1–9.8) RA: 5.9 min (3.2–9.5) Total duration of surgery (Mean + 95% CI): Manual: 46.9 min (33.0–60.8) RA: 42.7 min (26.0–59.3)	–	–	Operating microscope (Zeiss Rescan 700; Carl Zeiss Meditec AG) with iOCT (Zeiss Resight 700)
Yang et al	Manual: 82.2 s RA: 254.4 s	Mean motion amplitude: Manual: 18.8779 pixels RA: 0.3681 pixels	–	Operating microscope (Leica) with OCT (OCT-HS100, Canon)
Maierhofer et al	Manual: 32 s ± 17 s RA: 120 s ± 65 s	–	–	Operating microscope with OCT (OPMI LUMERA 700 with RESCAN 700, Carl Zeiss Meditec AG)

### Robots

Three different robotic setups were used for the SI [9, 15]. Preceyes system is controlled with the use of handheld motion controller with clutch and joystick. The robot from Maierhofer is controlled with the use of 3D mouse, while the RASR uses joystick. In terms of safety features, Preceyes system offers virtual boundary and tremor filtering, while RASR has instrument stabilization feature. While being relatively simple construction, the robotic system from Maierhofer study requires the steepest learning curve, due to the very specific control. RASR potentially offers (according to the technical specifications, which were not tested in detail) highest accuracy of all systems, while Preceyes offers balance between functionality and ease of use (steering).

### In-human SI

Kapetanovic et al. conducted a pioneering double-armed RCT to evaluate the efficacy of RA SI drug delivery in patients with submacular hemorrhage [12]. To the best of our knowledge, this is the only trial reported in literature assessing in-human RA SI. The RCT recruited 12 patients, which underwent subretinal injection of a TPA 1 mg/mL solution—six manually and six with robot assistance. The authors noted that vitreoretinal surgeons and staff received comprehensive training prior to the RA surgery, but the duration was not specified. Three port pars plane vitrectomy was used among the patients. Intraoperative optical coherence tomography (iOCT) provided visual feedback during the procedures. Once the needle was positioned within the macular hemorrhage at a distance of 100 µm from the retina, the RA mode was switched to step, and the needle was advanced further into the retina in increments of 20–30 µm.

The procedure was well-tolerated by all participants; however, one RA case required manual conversion due to a posterior subcapsular cataract obscuring the needle’s view. The median number of retinotomies and the volume of TPA injected did not differ significantly between the RA and manual groups (*p* = 0.34 and *p* = 0.31, respectively). The median subretinal injection time was comparable—5.9 min for RA and 4.3 min for manual (*p* = 0.93). No significant differences were observed in the total duration of surgery (RA = 46.9 min vs. manual = 42.7 min, *p* = 0.61), nor in the incidence of retinal microtrauma. The median visual acuity gain was 1.62 logMAR for the RA group versus 1.30 for the manual group (*p* = 0.14), with no robotic malfunctions reported during the trial.

### Ex vivo SI

Yang et al. evaluated the efficacy of a custom robot (RASR) from Beihang University on porcine eyes [14]. Five eyes were used in both the manual and RA groups, with a simulation involving the delivery of a 1% sodium fluorescein solution for 30 s. The formation of retinal bleed or entry into the vitreous cavity was monitored during injection. Triple channel conventional planar vitrectomy approach was utilized in the study. Conventional OCT was used for the visualization instead of iOCT.

Both groups achieved a 100% success rate. The subretinal area in RA group was not statistically significant different from manual group (*p* > 0.05), like the volume of the subretinal fluid (*p* > 0.05). Tremor motion of the needle was analyzed from recorded videos and measured in pixels. The RA group exhibited a statistically lower mean motion amplitude compared to the manual group (0.3681 vs. 18.8779 pixels, *p* < 0.0001). The mean operative time was longer in the RA group (4.24 vs. 1.37 min).

Maierhofer et al. utilized 72 porcine eyes to compare the efficacy of their custom robot with manual surgery, simulating drug injection with perfluorocarbon liquid [13]. Standard triple channel pars plana vitrectomy approach was performed. OPMI LUMERA 700 with Carl Zeiss RESCAN 700 was used for real-time visualization.

Successful blister formation was higher in the RA group (73.7% vs. 61.8%). Ten unsuccessful RA operations and thirteen manual ones were attributed to reflux, RPE damage, and rupture of the subretinal bleb. No significant differences in complications were found between the two groups (*p* = 0.279). The incidence of reflux was lower in the RA group (*p* < 0.01), as was the case in successful SIs (14.3% vs. 66.7%, *p* < 0.001). The average surgery duration was longer for RA (120 s vs. 32 s, *p* < 0.001), and RPE penetration rates did not differ significantly (*p* = 0.079).

### Artificial eye SI

Ladha et al. conducted subretinal injection surgeries using the Preceyes robotic system on a gelatin artificial eye model, which simulated a gene therapy setup [5]. The model’s retina had 2 layers: the top layer represented the neurosensory retina, and the bottom layer represented the RPE, choroid, and sclera. Openings were made to mimic the limbus edge and sclerotomy sites for subretinal injections. OPMI LUMERA 700 surgical microscope with Carl Zeiss Resight iOCT system were applied for visualization of the procedure, and ink mixture was used for infusion simulation. The experiments took place at the Paris Euretina 2019 Congress, with nine vitreoretinal surgeons of varying experience levels participating. Each surgeon received a brief training on the robotic system before practicing for 5 min. They were then randomly assigned to start with either the robot-assisted or manual method. The task involved inserting the needle to the required depth, starting the injection pump connected to the needle, and maintaining the needle's position for 60 s or until a bleb was visible beneath the top layer.

In manual subretinal injections, a bleb formed in 4 out of 9 cases, and reflux occurred in 8 out of 9 cases. With robot-assisted subretinal injections, a bleb formed in 8 out of 9 cases, and reflux occurred in 7 out of 9 surgeries. Puncture hole enlargement was not observed in robot-assisted cases but occurred in 7 out of 9 manual cases. The median injection time was longer for robot-assisted procedures (52 s) compared to manual (29 s). However, the median tremor was lower in the robot-assisted group (1 µm vs. 18 µm), as was the median drift (16 µm vs. 213 µm).

## Discussion

Subretinal injection is being more commonly used in the field of ophthalmology. The first subretinally injected drug was Voretigen Neparvovec (Luxturna), which was approved by American Food and Drug Administration in the 2017 [16]. This medicament was used as therapy for RPE65-mediated retinal dystrophy – being untreated, it would progress into the completed blindness. Initial trials showed promise into improvement of functional vision, which was before untreatable [16]. Compared to intravitreal or suprachoroidal injections, SI allows for drug delivery directly into retina cells, where intravitreal injections rely on diffusion from intravitreal cavity, resulting in lower absorption due to the presence of impermeable internal limiting membrane [17–20]

This systematic review explored the potential of robot-assisted subretinal injection (RA SI) as a drug delivery method for retinal diseases. We aimed not only to assess the efficacy of the RA procedures, but additionally provide limitations of such devices, future directions, and advantages compared to manual techniques. While the technology is still in its early stages, the findings suggest promising advantages over traditional manual procedures.

RA SI demonstrates significant tremor reduction compared to manual techniques, as shown in studies with porcine eyes and artificial models [5, 14]. This translates to a potentially lower risk of complications such as retinal tears, choroidal penetration, and macular holes.

Studies using porcine eyes also indicated potential reduction in subretinal reflux, a complication where the injected drug leaks back out [13]. Included studies suggest that RA SI may improve drug delivery efficiency.

The first-in-human randomized controlled trial (RCT) by Kapetanovic et al. yielded encouraging results [12]. While visual acuity improvement did not reach statistical significance, the procedure was well tolerated, and there were no major differences in surgical times or complications between RA SI and manual groups. Ex vivo studies using porcine eyes also achieved successful drug delivery in both RA and manual groups [13, 14].

Robotic devices offer many novel features, which are unachievable for surgeons due to the human limitations. For example, the Preceyes robotic system has a standby mode, which allows the needle to remain in stabilized position during the subretinal injection process up to 20 min. Achieving such position by humans is impossible due to naturally occurring tremor. This allows for stable gene therapy process, which require slow delivery infusion. Additionally, lower vector dose may be delivered through a larger area requiring therapy, increasing rate of the absorption of the drug, and minimizing the risk of occurrence of the retinal detachment. Lowering the infusion rate of the drug delivery opens a gate for more precise monitoring of the drug delivery process—intraoperative OCT applied in majority of the studies included in this systematic review allows to accurate real-time monitoring of the needle injection and pressure, lowering the chance of the foveal stretch and subretinal damage to the tissue [21].

When speaking about the iOCT, additionally, the fact that retina is a transparent tissue limits the depth assessment of the needle through the microscope during the surgery, due to low illumination. Too deep injection may result in irreversible damage to the RPE layer, and rupture of blood vessels in the area. On the other hand, too shallow injection will not effectively distribute the drug into subretinal area, lowering the absorption rate, as well as effectiveness of the SI.

Robotic devices may especially be beneficial in surgical procedures, where entering the same area (retinotomy) is required multiple times in an operation (multi-staged gene therapies). A feature used in the Preceyes System is the return to position, which allows to return to exactly the same former position [22]. Chance of vector reflux, inflammation risk, and reduction of retinotomy size would be lowered in such cases. In case of critical emergency during the SI, robot can eject intraocular instrument in about half of the second.

Currently, RA SI procedures take longer than manual techniques [5, 13, 14]. This might be due to the novelty of the technology and the learning curve for surgeons—robots contribute their operation time for maximum accuracy of the injection, which requires additional procedure time for more controlled movements of the system. Additionally, robots require proper setup before the operation, which also increases total procedure time [23]. Further development and comprehensive training could potentially shorten these times. The current body of research is also limited, with only one RCT and a handful of ex vivo studies. More extensive clinical trials are needed to definitively assess the safety and efficacy of RA SI compared to established techniques.

Advancements in robotic technology, including improved dexterity and miniaturization of instruments, could further enhance the capabilities of RA SI. Integration of real-time feedback mechanisms, such as haptic technology and augmented reality, could improve surgeon control and decision-making during RA SI procedures. Implementation of Artificial Intelligence (AI) could potentially allow for semi/fully autonomous robotic eye surgery, when coupled with OCT imaging, and surgery planning. For example, intraoperative 4D OCT with Machine Learning algorithm, is being tested for subretinal injection surgery [24]. Autonomous robotic navigation systems, which utilize deep learning techniques are being more frequently tested in the literature, showing potential promise in performing surgeries without any human intervention [25–27]. These convolutional networks, which segment tip of the needle in the real time, allow for potentially even greater precision and control, compared to the conventional machine learning algorithms. Finally, in the early stages of the robot development, instead of using ex vivo porcine eyes, experiments should be tested on human donor eyes, to simulate more realistically surgical operations.

The full integration of robotic systems into SI procedures will require overcoming all mentioned limitations. Additionally, the cost-effectiveness of the devices must be analyzed, as none of the studies have provided or fully analyzed this aspect. As robots could reduce the potential complications, this would decrease the number of additional procedures, leading to reduced costs. However, the implementation price of the robotic system into surgical room must be taken into account, and analyzed if it requires specific conditions to be proven to be cost-effective. Further development of commercially available surgical systems for eye interventions could allow to choose the most suitable one, as currently market is very limited, and may not be accessible in some countries. Comprehensive surgical training programs would enhance the effectiveness for current, and upcoming operations of such robotic devices. Last, but not least the more in-human interventions are required, to further analyze novel technological solutions, which are in majority tested on porcine models.

## Conclusions

Subretinal drug injection procedures are among the most precise and demanding in ophthalmology, requiring exact placement of medication directly under the retina. The development of robots in this field, as in other areas of surgery, will be linked to potential practical benefits and the possibility of introducing automation and robot autonomy in the future. The specifics of using robots in these procedures are connected with:

1. Precision in localization
2. Stability and accuracy
3. Microscale operations
4. Depth control

Their potential advantages over manual approaches are caused by:

1. Reduction of human errors
2. Improved clinical outcomes
3. Reduced procedure time
4. Learning and adaptation capabilities

Robotic systems driven by AI can learn from each operation performed, continually refining their algorithms for better adjustment to the anatomical conditions of different patients. However, potential challenges include cost and availability, as well as training curve for medical staff.

Robot-assisted subretinal injection holds promise for revolutionizing intraocular surgeries. While still in its very early stages, preliminary data suggests RA SI offers advantages in terms of precision, reduced complications, and potentially improved drug delivery efficiency. Further research and development are crucial to refine this technology and establish its role in clinical practice.

## Data Availability

No datasets were generated or analyzed during the current study.

## Appendix—Search strategy

**PubMed**: 19—(robot OR robot-assisted) AND (subretinal injection OR subretinal drug delivery).

**Embase**: 16—('robot'/exp OR robot OR 'robot assisted') AND ('subretinal injection'/exp OR 'subretinal injection' OR (subretinal AND ('injection'/exp OR injection)) OR 'subretinal drug delivery' OR (subretinal AND ('drug'/exp OR drug) AND ('delivery'/exp OR delivery))).

**Scopus**: 5—( robot OR robot-assisted) AND ( subretinal AND injection OR subretinal AND drug AND delivery).

**Web of Science**: 18—(robot OR robot-assisted) AND (subretinal injection OR subretinal drug delivery).

**Cochrane Reviews**: 5—(robot OR robot-assisted) AND (subretinal injection OR subretinal drug delivery).

## Abbreviations

RA: Robot assisted
SI: Subretinal injection
LogMAR: Logarithm of minimal angle of resolution

## References

[1] Davis JL, Gregori NZ, MacLaren RE, Lam BL (2019) Surgical technique for subretinal gene therapy in humans with inherited retinal degeneration. Retina 39(1):S2-8 31335483 10.1097/IAE.0000000000002609

[2] Holtkamp GM, Kijlstra A, Peek R, de Vos AF (2001) Retinal pigment epithelium-immune system interactions: cytokine production and cytokine-induced changes. Prog Retin Eye Res 20(1):29–48 11070367 10.1016/s1350-9462(00)00017-3

[3] Diniz B, Thomas P, Thomas B, Ribeiro R, Hu Y, Brant R et al (2013) Subretinal implantation of retinal pigment epithelial cells derived from human embryonic stem cells: improved survival when implanted as a monolayer. Investig Opthalmol Visual Sci 54(7):5087 10.1167/iovs.12-11239 PMC3726243 23833067

[4] Khristov V, Maminishkis A, Amaral J, Rising A, Bharti K, Miller S (2018) Validation of iPS cell-derived RPE tissue in animal models. Adv Exp Med Biol. 10.1007/978-3-319-75402-4_77 29721997 10.1007/978-3-319-75402-4_77 PMC8783981

[5] Ladha R, Meenink T, Smit J, de Smet MD (2021) Advantages of robotic assistance over a manual approach in simulated subretinal injections and its relevance for gene therapy. Gene Ther. 10.1038/s41434-021-00262-w 34002047 10.1038/s41434-021-00262-w PMC10113148

[6] G Yiu, SH Chung, IN Mollhoff, UT Nguyen, SM Thomasy, J Yoo et al. 2020 Suprachoroidal and subretinal injections of aav using transscleral microneedles for retinal gene delivery in nonhuman primates. Mole Therapy — Methods Clinic Develop. 16:179–91. https://www.ncbi.nlm.nih.gov/pmc/articles/PMC7005511/pdf/main.pdf. Accessed 3 Mar 2024 10.1016/j.omtm.2020.01.002 PMC7005511 32055646

[7] K Ding, J Shen, Z Hafiz, SF Hackett, RL Silva e, M Khan et al. 2019 AAV8-vectored suprachoroidal gene transfer produces widespread ocular transgene expression. J Clinic Invest. 129:(11) 4901–11. https://www.jci.org/articles/view/129085. Accessed 3 Mar 2024 10.1172/JCI129085 PMC6819121 31408444

[8] de Smet MD, Naus GJL, Faridpooya K, Mura M (2018) Robotic-assisted surgery in ophthalmology. Curr Opin Ophthalmol 29(3):248–253 29553953 10.1097/ICU.0000000000000476

[9] de Smet MD, Meenink TCM, Janssens T, Vanheukelom V, Naus GJL, Beelen MJ et al (2016) Robotic assisted cannulation of occluded retinal veins. PLoS ONE 11(9):0162037 10.1371/journal.pone.0162037 PMC5046264 27676261

[10] PRECEYES Surgical System – Preceyes BV. https://www.preceyes.nl/preceyes-surgical-system/. Accessed 3 Mar 2024

[11] MJ Page, JE McKenzie, PM Bossuyt, I Boutron, TC Hoffmann, CD Mulrow et al. 2021 The PRISMA 2020 statement: an updated guideline for reporting systematic reviews. British Medi J 29;372(71). https://www.bmj.com/content/372/bmj.n71. Accessed 3 Mar 2024 10.1136/bmj.n71 PMC8005924 33782057

[12] J Cehajic-Kapetanovic, K Xue, TL Edwards, TC Meenink , MJ Beelen, GJ Naus et al. 2021 First-in-human robot-assisted subretinal drug delivery under local anaesthesia a randomised clinical trial. Am J Ophthalmol. https://www.sciencedirect.com/science/article/abs/pii/S0002939421005924. Accessed 3 Mar 2024 10.1016/j.ajo.2021.11.011 34788592

[13] Maierhofer NA, Jablonka AM, Hessam Roodaki M, Nasseri A, Eslami A, Klaas J et al (2023) iOCT-guided simulated subretinal injections: a comparison between manual and robot-assisted techniques in an ex-vivo porcine model. J Robot Surg 17(6):2735–2742 37670151 10.1007/s11701-023-01699-4 PMC10678791

[14] Yang K, Jin X, Wang Z, Fang Y, Li Z, Yang Z et al (2022) Robot-assisted subretinal injection system: development and preliminary verification. BMC Ophthalmol. 10.1186/s12886-022-02720-4 36510151 10.1186/s12886-022-02720-4 PMC9744060

[15] Nasseri MA, Maier M, Lohmann CP (2017) A targeted drug delivery platform for assisting retinal surgeons for treating age-related macular degeneration (AMD). PubMed 10.1109/EMBC.2017.8037815 29060856

[16] Pennesi ME, Schlecther CL (2020) The evolution of retinal gene therapy: from clinical trials to clinical practice. Ophthalmology 127(2):148–150 31973830 10.1016/j.ophtha.2019.12.003

[17] Peng Y, Tang L, Zhou Y (2017) Subretinal injection: a review on the novel route of therapeutic delivery for vitreoretinal diseases. Ophthalmic Res 58(4):217–226 28858866 10.1159/000479157

[18] Dalkara D, Kolstad KD, Caporale N, Visel M, Klimczak RR, Schaffer DV et al (2009) Inner limiting membrane barriers to aav-mediated retinal transduction from the vitreous. Mol Ther 17(12):2096–2102 19672248 10.1038/mt.2009.181 PMC2814392

[19] GA Ochakovski, KU Bartz-Schmidt, MD Fischer. 2017 Retinal gene therapy: surgical vector delivery in the translation to clinical trials. Front Neurosci. https://www.ncbi.nlm.nih.gov/pmc/articles/PMC5376580/. Accessed 3 Mar 2024 10.3389/fnins.2017.00174 PMC5376580 28420956

[20] Gregori NZ, Lam BL, Davis JL (2019) Intraoperative use of microscope-integrated optical coherence tomography for subretinal gene therapy delivery. Retina 39(1):S9-12 28426632 10.1097/IAE.0000000000001646

[21] Zhou M, Huang K, Eslami A, Roodaki H, Zapp D, Maier M et al (2018) Precision needle tip localization using optical coherence tomography images for subretinal injection. mediaTUM (Technical University of Munich)

[22] TL Edwards, K Xue, HCM Meenink, MJ Beelen, GJL Naus, MP Simunovic et al. 2018 First-in-human study of the safety and viability of intraocular robotic surgery. Nat Biomed Eng 2(9):649–56. https://www.nature.com/articles/s41551-018-0248-4. Accessed 18 May 2024 10.1038/s41551-018-0248-4 PMC6155489 30263872

[23] Faridpooya K, Romunde van, Manning SS, Jan, Naus L, Beelen MJ, et al (2022) Randomised controlled trial on robot-assisted versus manual surgery for pucker peeling. Clin Experiment Ophthalmol 50(9):1057–1064 36177965 10.1111/ceo.14174

[24] Sommersperger M, Weiss J, Ali Nasseri M, Gehlbach P, Iordachita I, Navab N (2021) Real-time tool to layer distance estimation for robotic subretinal injection using intraoperative 4D OCT. Biomed Opt Express 12(2):1085–1095 33680560 10.1364/BOE.415477 PMC7901333

[25] Dehghani S, Sommersperger M, Zhang P, Martin-Gomez A, Busam B, Gehlbach P et al (2023) Robotic navigation autonomy for subretinal injection via intelligent real-time virtual iOCT, vol Slicing. Arxiv (Cornell University), NY 10.1109/icra48891.2023.10160372 PMC10732544 38125032

[26] Zhang P, Kim JW, Gehlbach P, Iordachita I, Kobilarov M (2024) Autonomous needle navigation in subretinal injections via iOCT. IEEE Robotics & Automat Letters 9(5):4154–4161 10.1109/lra.2024.3375710 PMC10972538 38550718

[27] Kim JW, Wei S, Zhang P, Gehlbach P, Kang JU, Iordachita I et al (2024) Towards autonomous retinal microsurgery using RGB-D Images. IEEE Robot and Automat Letters 10.1109/lra.2024.3368192 PMC11415253 39309968

