# Correction: Sharper vision, steady hands: can robots improve subretinal drug delivery? Systematic review

**DOI:** 10.1007/s11701-026-03409-2

**Published:** 2026-04-18

**Authors:** Paweł Marek Łajczak, Zbigniew Nawrat

**Affiliations:** 1 Zbigniew Religa Student Scientific Club at Department of Biophysics, Faculty of Medical Sciences in Zabrze, Medical University of Silesia in Katowice, Jordana 18, 40-043 Zabrze, Poland; 2 Department of Biophysics, Faculty of Medical Sciences in Zabrze, Medical University of Silesia in Katowice, Jordana 18, 40-043 Zabrze, Poland; 3 Foundation of Cardiac Surgery Development, 41-808 Zabrze, Poland

**Correction: Journal of Robotic Surgery (2024) 18:235**

10.1007/s11701-024-01991-x

Corrigendum to "Sharper Vision, Steady Hands: Can Robots Improve Subretinal Drug Delivery? Systematic Review"

The authors acknowledge that in the originally published version of this article, the Declaration of Generative AI and AI-assisted Technologies in the Writing Process was inadvertently omitted. This oversight has now been corrected, and the declaration has been included. The authors apologize for any inconvenience caused.

Declaration of Generative AI and AI-assisted technologies in the writing process: During the preparation of this work, the authors used Gemini (Google Inc.) during writing and grammar checking process. After using this tool, the authors reviewed and edited the content as needed and take full responsibility for the content of the published article. A large language model was not used for data extraction, screening, or statistical analysis. All ideas, analysis, and final content were produced and approved by the authors.

